# Anoikis-resistant subpopulations of human osteosarcoma display significant chemoresistance and are sensitive to targeted epigenetic therapies predicted by expression profiling

**DOI:** 10.1186/s12967-015-0466-4

**Published:** 2015-04-02

**Authors:** Jessica M Foley, Donald J Scholten II, Noel R Monks, David Cherba, David J Monsma, Paula Davidson, Dawna Dylewski, Karl Dykema, Mary E Winn, Matthew R Steensma

**Affiliations:** Helen DeVos Children’s Hospital, Spectrum Health System, Grand Rapids, MI USA; Van Andel Research Institute, Grand Rapids, MI USA; Michigan State University College of Human Medicine, Grand Rapids, MI USA

**Keywords:** Anchorage-independent growth, Osteosarcoma, Anoikis resistance, Expression profiling

## Abstract

**Background:**

Osteosarcoma (OS) is the most common type of solid bone cancer, with latent metastasis being a typical mode of disease progression and a major contributor to poor prognosis. For this to occur, cells must resist anoikis and be able to recapitulate tumorigenesis in a foreign microenvironment. Finding novel approaches to treat osteosarcoma and target those cell subpopulations that possess the ability to resist anoikis and contribute to metastatic disease is imperative. Here we investigate anchorage-independent (AI) cell growth as a model to better characterize anoikis resistance in human osteosarcoma while using an expression profiling approach to identify and test targetable signaling pathways.

**Methods:**

Established human OS cell lines and patient-derived human OS cell isolates were subjected to growth in either adherent or AI conditions using Ultra-Low Attachment plates in identical media conditions. Growth rate was assessed using cell doubling times and chemoresistance was assessed by determining cell viability in response to a serial dilution of either doxorubicin or cisplatin. Gene expression differences were examined using quantitative reverse-transcription PCR and microarray with principal component and pathway analysis. *In-vivo* OS xenografts were generated by either subcutaneous or intratibial injection of adherent or AI human OS cells into athymic nude mice. Statistical significance was determined using student’s t-tests with significance set at α = 0.05.

**Results:**

We show that AI growth results in a global gene expression profile change accompanied by significant chemoresistance (up to 75 fold, p < 0.05). AI cells demonstrate alteration of key mediators of mesenchymal differentiation (β-catenin, Runx2), stemness (Sox2), proliferation (c-myc, Akt), and epigenetic regulation (HDAC class 1). AI cells were equally tumorigenic as their adherent counterparts, but showed a significantly decreased rate of growth *in-vitro* and *in-vivo* (p < 0.05). Treatment with the pan-histone deacetylase inhibitor vorinostat and the DNA methyltransferase inhibitor 5-azacytidine mitigated AI growth, while 5-azacytidine sensitized anoikis-resistant cells to doxorubicin (p < 0.05).

**Conclusions:**

These data demonstrate remarkable plasticity in anoikis-resistant human osteosarcoma subpopulations accompanied by a rapid development of chemoresistance and altered growth rates mirroring the early stages of latent metastasis. Targeting epigenetic regulation of this process may be a viable therapeutic strategy.

**Electronic supplementary material:**

The online version of this article (doi:10.1186/s12967-015-0466-4) contains supplementary material, which is available to authorized users.

## Introduction

Osteosarcoma (OS) is the most common type of solid bone cancer, mainly arising in children and young adults. The long term survival for non-metastatic presentations of OS is 70%, but recurrence continues to be associated with an extremely poor prognosis [1]. The survival rate for recurrent osteosarcoma is less than 30%, and has remained relatively constant since the initiation of doxorubicin-based chemotherapy regimens in the 1980’s [1]. Before routine use of chemotherapy, amputation was typically the treatment for patients with localized osteosarcoma, but the 5-year survival following amputation was only 17% [2], confirming by natural history that hematogeneous dissemination occurs early in the disease process. Even today, with modern staging methods, micrometastatic disease is not readily detectable [3].

Anchorage-independent (AI) growth is an essential feature of malignancy [4] and metastatic progression [5]. The cancer cells must avoid anoikis, i.e., programmed cell death in the absence of attachment to extracellular matrix, for this to take place [6-8]. Anoikis is known to occur through a Bcl-2-mediated intrinsic pathway [9,10] or through an alternative extrinsic pathway in which Apo1/Fas or TRAIL (tumor necrosis factor-related apoptosis-inducing ligand) binding to cell-surface death receptors triggers apoptosis [11]. AI growth has been associated with disease progression and metastatic potential in breast cancer, lung cancer, and melanoma [7]. Anoikis resistance in epithelial-lines has been associated with chemotherapy resistance and enhanced tumorigenicity [12-14], and increased expression of markers – such as Oct4, Nanog, and Sox2 – that are important in stem cell maintenance and development [12,13,15,16]. Many of these markers are known to facilitate the oncogenic epithelial-to-mesenchymal transition [17,18].

Much work using AI cell growth has been coupled with manipulation of growth factor concentrations to promote an undifferentiated and cancer stem cell-like state, without paying close attention to processes driving a cell’s ability to resist anoikis, and the phenotypic consequences of those processes. Using a model of AI cell growth, we sought to characterize the functional consequences of anoikis resistance in human osteosarcoma, particularly as they relate to the promotion of therapy resistance and recurrence, while predicting and validating novel therapies to target these cell subpopulations. We demonstrate that AI growth of established and patient-derived osteosarcoma cells alters the cellular growth rate and promotes chemotherapy resistance, while AI induced changes in growth rate persisted *in-vivo*. AI growth resulted in a rapid change in the gene expression profile including upregulation of the stem cell factor, sex determining region Y-box 2 (Sox2). c-Myc, β-catenin, Akt, and histone deacetylases were confirmed to be a central part of the AI growth molecular signature. Lastly, treatment with the pan-histone deacetylase inhibitor vorinostat and the DNA methyltransferase inhibitor 5-azacytidine decreased AI cell growth, while 5-azacytidine sensitized anoikis-resistant cells to doxorubicin. These data highlight the importance of epigenetic regulation in anoikis resistance and altered cell growth during osteosarcoma disease progression.

## Methods

### Anchorage-independent culture technique

The human OS cell lines were derived from consenting OS patients according to protocols approved by the local ethics committee (IRB full name: Spectrum Health Institutional Review Board; part of the Spectrum Health Research Protection Program). Written informed consent was obtained from the human research subjects under an IRB-approved (IRB full name: Spectrum Health Institutional Review Board), musculoskeletal tumor and tissue acquisition protocol at Spectrum Health (2011-002). Osteosarcoma cell lines 143B, MNNG/HOS (mHOS), and MG-63 (ATCC) were cultured in Ultra-Low Attachment plates (Corning) using DMEM or MEM (Gibco) supplemented with 1% L-glutamine, 1% penicillin/streptomycin, and 10% fetal bovine serum (FBS) (Gibco). Cells isolated from chemo-naïve and post-chemotherapy patient tumor samples were also grown in these Ultra-Low-Attachment plates in the presence of MEMα (Gibco) with 1% L-glutamine, 1% penicillin/streptomycin, and 10% FBS. Following 4-6 days of growth, the cells were passaged using 0.05% trypsin (Gibco) dissociation and re-plated in Ultra-Low Attachment plates. With each passage, viabilities of single cells were assessed using trypan blue; mean and standard error were calculated using Microsoft Excel. The determination of cell doublings per day was calculated using the following formula:

$$ \mathrm{Doublings}/\mathrm{day}\kern0.5em =\kern0.5em \frac{ \log 2\kern0.5em \left(\mathrm{Final}\kern0.5em \mathrm{Cell}\kern0.5em \mathrm{Number}\kern0.5em \div \kern0.5em \mathrm{Starting}\kern0.5em \mathrm{Cell}\kern0.5em \mathrm{Number}\right)}{\mathrm{Number}\kern0.5em \mathrm{of}\kern0.5em \mathrm{Days}} $$

Statistical significance was determined using a two-tailed two-sample *t*-test using R with significance set at α = 0.05 [19].

### Quantitative reverse transcription-PCR (qrt-PCR)

Total RNA was isolated using TRIzol reagent (Invitrogen). Complementary DNA synthesis was performed using 500 ng RNA according to the instructions with the High Capacity cDNA Reverse Transcription Kit (Invitrogen). Primer sequences for human genes of interest are as follows (shown here, 5′ to 3′): Oct4 primers (F:TAAGCTGCGGCCCTTGCTGC; R:CGGGCCTGCACGAGGGTTTC), Sox2 primers (F:GGGGAAAGTAGTTTGCTGCCTCTT; R:TGCCGCCGCCGATGATTGT), Axin2 primers (F:AAGCAAGCGATGAGTTTGCCTGTG; R:ACAGCCAAGACAGTTCACAAGAGC), GAPDH primers (F:AACTTTGGCATTGTGGAAGG; R:GGATGCAGGGATGATGTTCT). Quantitative polymerase chain reactions were performed using SYBR Select Mastermix (Applied Biosystems, Carlsbad, CA) in 10 μL reactions. Polymerase chain reaction was performed according to the manufacturer’s instructions using a StepOnePlus cycler (Applied Biosystems). Cross-threshold (C_T_) values were calculated using the version 2.1 software (Applied Biosystems). Data were analyzed using the 2^-ΔΔC^_T_ method [20]. Data were presented as mean and standard deviation using Microsoft Excel, with statistical significance being determined using a two-tailed, one-sample *t*-test with μ = 1 using R and significance set at α = 0.05 [19].

### Western blot analysis

Cells were washed with PBS and lysed on ice in lysis buffer (RIPA lysis buffer) supplemented with complete Protease Inhibitor Cocktail (Roche). Protein concentration was measured using the BCA assay (Pierce), and 20 μg of whole-cell lysate was run on a 10% SDS/polyacrylamide gel. The proteins were transferred onto a nitrocellulose membrane, and membranes were probed overnight at 4°C with the appropriate primary antibody; antibodies used were as follows: active β-catenin, β-catenin, Runx2 (Cell Signaling Technologies); Ki67 (Spring Bioscience); and Actin (Millipore). Membranes were then probed with a horseradish-peroxidase-conjugated secondary antibody for 1.5 hours at room temperature before detection using an enhanced chemiluminescence (ECL) detection system (Pierce). Because western blot analysis was used to support microarray and cell growth findings, images shown are representative of one independent experiment for each cell line depicted.

### Gene expression profiling and analysis

RNA was isolated as described above from established (n = 3) and patient-derived (n = 7) human osteosarcoma cell lines that were cultured in adherent or anchorage-independent conditions. Total RNA was prepared separately from each individual cell line grown under adherent or anchorage-independent conditions. Established cell lines used included 143B, MNNG/HOS, and MG-63, while patient-derived cell lines included MS124, MS124-2, MS145, MS145-2, MS206, MS206-2, and MS088. Human U133 Plus 2.0 arrays were performed at Clinical Reference Laboratories (CRL, Lenexa, KS). Purified RNA (5 ng) was used for amplification of cDNA (NuGen Ovation Pico WTA System). cDNA was then fragmented and labeled (NuGen Encore Biotin Module) and hybridized to GeneChip Human Genome U133 Plus 2.0 Array (GeneChip Hybridization, Wash and Stain Kit, Affymetrix). Standard quality control metrics for percent present, scale factor, and RNA quality were evaluated for each sample. Principal component analysis (PCA) was completed using an R (ver 3.10) workflow using the BioConductor *affy* (ver 1.40.0) package for RMA normalization and the “prcomp” function from the *stats* package. Two analysis approaches were taken for differential expression analysis. Approach 1: Affymetrix CEL files for both patient-derived and established cell lines were processed with Affymetrix Expression Console using MAS5.0 normalization for the differential expressed top 300 gene list using a Welch’s T-test applied to log base 2 transformed data. The top 300 genes were imported into MetaCore from Thomson Reuters (version 6.19 build 65960) for pathway and network analysis. The top two ranked pathways identified by the *Analyze network* feature are shown in Additional file 1: Figure S1a and b. The *Shortest paths* feature with length = 1 and canonical pathways disabled was used for shortest pathway analysis. The top 300 genes are supplied in Additional file 2: Table S1, split into upregulated and downregulated groups ordered by t-statistic value. No false discovery rate correction was used because the intended purpose of the gene list was for a discovery investigation of pathways utilizing the GeneGo database. Additional file 1: Figure S1a and b shows an interaction network captured using MetaCore derived from a significant gene list. The lines that connect the gene symbols on the MetaCore image represent the direction of interaction and the type of interaction. The arrow points to the gene that is affected and the type of interaction is indicated by the color of the line. Lines with color red means inhibition, green means activation, and grey indicates an unspecified type of interaction. The concentric circles with red centers show that the gene was in the gene list and up regulated. The concentric circles with blue indicates the gene was in the gene list and was down regulated. The various gene symbols represent classes of gene types. Generic binding genes are blue “S” shaped, proteins are shown as three filled blue circles overlapping, gold arrow shapes indicate generic kinase genes and gold arrows with a hole in the center indicate a generic protease. Transcription factors are shown in red with two points on top and three on the bottom. For the official legend refer to https://ftp.genego.com/files/MC_legend.pdf. Approach 2: Affymetrix CEL files for patient-derived cell lines were imported into Bioconductor/R for processing via 3 normalization procedures (RMA, FRMA, and MAS5.0 background correction; *affy* package) and differential expression analysis via paired *t*-test (*limma* package). Significantly altered genes were identified as those with p < 0.05 using a Benjamini & Hochberg false discovery rate correction [21] across the ensemble of normalization methods.

### Chemotherapy resistance assays

Passaged cells (minimum 2 passages) were dissociated and plated into 96-well Ultra Low Attachment plates (Corning) and allowed to grow for 4 days before chemotherapy exposure. Adherent cells were dissociated around 70% confluence. Cells were plated into 96-well white-walled plates (Greiner Bio-One) at 1 × 10^3^ cells/well and allowed to adhere for 24 hr before drug treatments. The cells were then exposed to one concentration from a serial dilution of doxorubicin (0-10 μM; LC Labs) or cisplatin (0-100 μM; Sigma) for 72 hr. Cell viability was then determined using the CellTiter-Glo Luminescent Cell Viability assay (Promega). Data were normalized to an untreated control well and graphed using GraphPad Prism software, and half maximal inhibitory concentration (IC_50_) values were calculated from the dose-response curve as the concentration of doxorubicin or cisplatin that produced a 50% decrease in the mean cell viability relative to untreated control wells. This is a standard method to assess resistance to chemotherapy-mediated toxicity in anchorage-independent cells as done in the literature [12,13]. Data normality was determined using the Shapiro-Wilk test. Statistical tests (two-tailed paired *t*-test and wilcoxon signed-rank test) were performed using R [19] with significance set at α = 0.05.

### Vorinostat and 5-azacytidine treatment

Adherent cells were dissociated around 70% confluence. Cells were plated into 96-well Ultra-Low Attachment plates (Corning) or adherent tissue culture plates at 1 × 10^3^ cells/well in the presence of 2 μM Vorinostat (LC Labs), with dimethyl sulfoxide (DMSO; Fischer) alone used as a control. Cell viability was assessed after 4 days using the CellTiter-Glo Luminescent Cell Viability assay (Promega). For chemoresistance assays, cells were treated after 24 hours with one concentration from a serial dilution of doxorubicin, with cell viability being measured after an additional 72 hours as described above. Pretreatment used either 2 μM Vorinostat or DMSO alone for 12 days in adherent conditions before plating the cells in 96-well Ultra Low Attachment plates or adherent plates. 5-azacytidine (5-azaC, Sigma) was dissolved in 50 mM sodium phosphate (vehicle), and adherent OS cells were treated with 2 μM 5-azaC or the same dilution of vehicle alone for 24 hours before being plated in 96-well Ultra-Low Attachment plates or adherent tissue culture plates in the absence of 5-azaC or vehicle. Cell viability or chemoresistance was then assessed as described above. Data were graphed using GraphPad Prism software, and two-tailed one-sample t-tests were performed using R with significance set at α = 0.05 [19].

### Orthotopic and non-orthotopic xenograft experiments

Following the approval of the research protocol by the IACUC committee at the Van Andel Institute (IACUC protocol #: 13-09-031 and XPA-14-02-004), cells subjected to either AI or adherent growth were dissociated into single-cell suspensions and were injected into the flank of or intratibially into athymic nude mice. For intratibial injection, mice were anesthetized via inhaled isoflurane and cells were injected in a total volume of 20 μL through the tibial plateau. All efforts were made to minimize suffering. Tumors were allowed to grow to 1500 mm^3^ or until euthanasia criteria were met. Tumor volume calculations of intratibial tumors were done using the formula V = (length × width^2^)/2 with the contralateral leg as a control. Volumes were statistically compared at each time point using a two-tailed two-sample *t*-test with significance set at α = 0.05.

## Results

### Anoikis-resistant OS cells robustly maintain viability and exhibit sustained but down-regulated rates of growth

**Figure 1:**
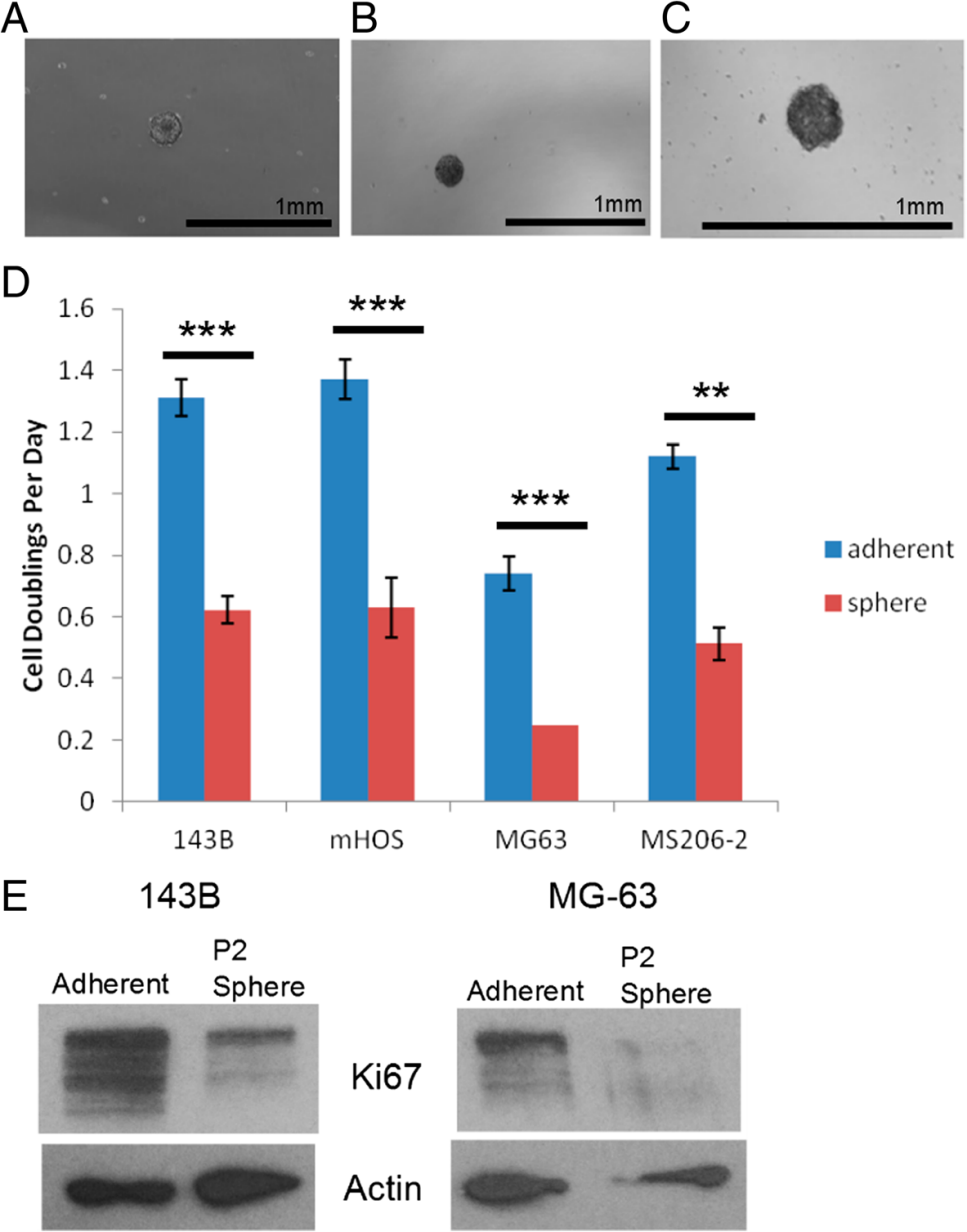
**Anoikis-resistant OS are viable and exhibit lower growth rates. A**-**C**: Representative AI-grown OS cells generated from mHOS **(A)**, 143B **(B)**, and a patient derived OS cell line, MS206-2 **(C)**. **D**: Growth rates measured as cell doublings per day. Results shown are average ± standard error. Asterisks indicate statistical significance (**p < 0.01, ***p < 0.001). **E**: Western blot analysis of 143B and MG-63 OS cells shows decreased Ki67 protein levels in AI grown cells (P2 spheres) compared to adherent cells.

When plated in ultra-low attachment plates, subpopulations of established and patient-derived human OS single cell suspensions generated spherical colonies within 4 days (Figure 1A-C). The viability of OS cells after continued growth in AI conditions remained high, comparable to that of adherent cells (data not shown). When we compared the growth rate of cells cultured in adherent conditions relative to those in AI conditions, cell doublings per day was 2-3 times greater for the adherent cells (Figure 1D). Prior studies have observed an increase in OS cell death in response to AI growth, which we also observed in some of our cell lines (data not shown) [22]. In cells surviving AI growth, we observed decreased Ki67 protein levels, indicating that proliferation differences can also contribute to these growth rate alterations (Figure 1E). Thus, subpopulations of human OS cells are capable of resisting anoikis and surviving AI growth, but at a lower growth rate.

### Anoikis-resistant OS cells have increased resistance to chemotherapy

**Figure 2:**
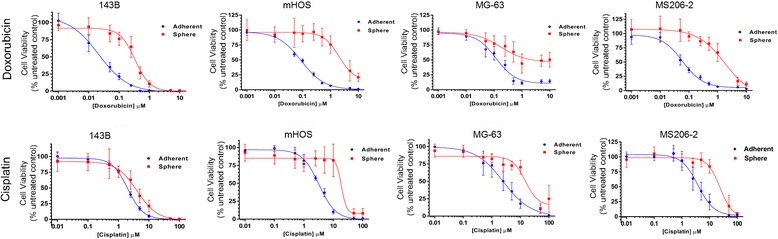
**Anoikis-resistant OS cells show resistance to standard OS chemotherapies.** Dose-response curves for cells grown under AI or adherent conditions treated with doxorubicin (top panel) or cisplatin (bottom panel) in a set of serial dilutions for 72 hours. Cell viability was determined as a percentage of the untreated control (0 μM doxorubicin or cisplatin).

To examine the effect of AI growth on the response of OS cells to chemotherapy, we treated both AI and adherent OS cells with increasing concentrations of mainstay OS chemotherapies (doxorubicin and cisplatin) and assessed cell viability after 72 hours. Across three established OS cell lines and a patient-derived cell line, AI cells showed a right shift in both dose-response curves (Figure 2). An average IC_50_ was calculated for each group (Table 1). In response to treatment with doxorubicin or cisplatin, the established OS cell lines 143B and mHOS as well as the patient-derived 206-2 OS cell line showed a statistically significant increase in IC_50_ (Table 1). We were not able to obtain an average IC_50_ for the MG-63 cell line due to an inability to achieve a consistent 50% growth inhibition in the AI group at the highest concentrations of chemotherapy tested.

**Table 1:** **72 hr IC**_**50**_ **for AI and adherent OS cells in the presence of doxorubicin or cisplatin**

		**Doxorubicin**		**Cisplatin**	
**Cell type**		**Mean IC50 (uM)**	**Fold change**	**Mean IC50 (uM)**	**Fold change**
MS206-2	Adherent	0.062 ± 0.01	28.8^	4.38 ± 0.98	5.7^
	AI	1.79 ± 0.4		25.2 ± 6.9	
143B	Adherent	0.025 ± 0.01	13*	1.97 ± 0.19	1.9*
	AI	0.31 ± 0.1		3.12 ± 0.10	
mHOS	Adherent	0.085 ± 0.013	35*	3.10 ± 0.53	6.9*
	AI	2.97 ± 1.9		21.4 ± 10.	
MG-63	Adherent	0.13 ± 0.06	>75#	2.68 ± 1.6	6.2*
	AI	>50% survival		16.9 ± 5.4	

^Indicates p < 0.05 (two-tailed two-sample *t*-test).

*Indicates p < 0.05 (wilcoxon signed-rank test).

#Indicates >50% cell viability after treatment, so we could not calculate an IC_50._

### Anoikis-resistant OS cells are molecularly distinct

**Figure 3:**
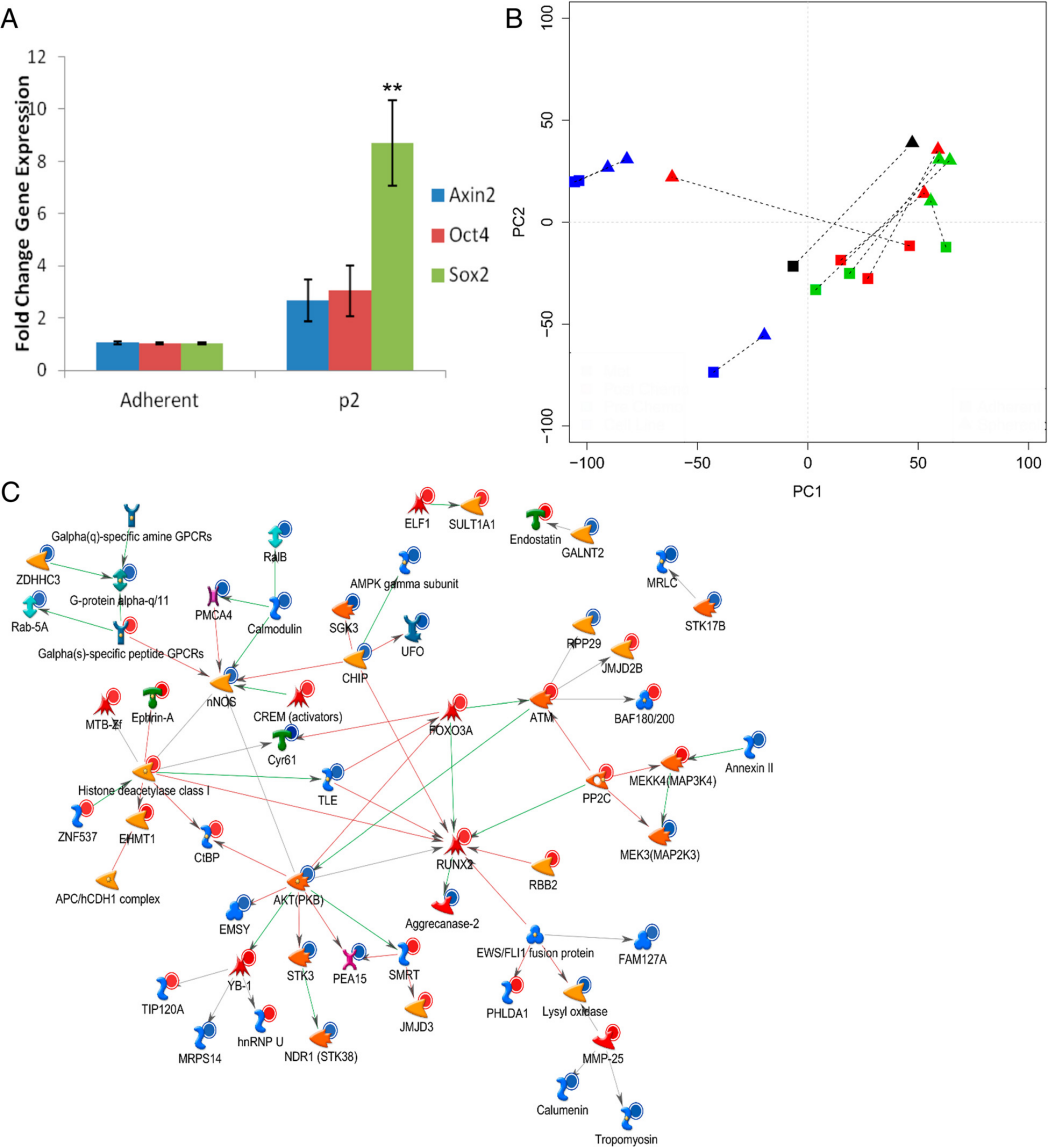
**Anoikis-resistant OS cells demonstrate distinct patterns of altered gene expression. A**: mRNA expression of the target genes Oct4, Axin2, and Sox2 in AI-grown OS cells after the second passage (p2) was determined using qrt-PCR normalized to adherent cells. Asterisks indicate statistical significance (**p < 0.01). **B**: Principal component analysis of gene expression using microarray. Gene expression differences resulted in clustering of the adherent cells (squares) compared to the AI cells (triangles). This was more apparent for the patient-derived OS cells than the established OS cell lines. Blue color indicates established OS cell line, green color indicates pre-chemotherapy patient-derived OS cell line, red color indicates post-chemotherapy patient-derived OS cell line, black color indicates pulmonary metastasis patient-derived OS cell line, dotted lines connect paired AI/adherent samples. **C**: Shortest pathway analysis of the top 300 differentially expressed genes identifies class I HDACs, Runx2, and Akt as central contributors.

We assessed gene expression differences that may be important for OS cell anoikis resistance using both qrt-PCR and microarray gene expression profiling. Expression of genes involved in developmental programs including Sox2, Oct4, and Wnt signaling were increased in the AI-grown cells relative to adherent cells (Figure 3A). This finding was statistically significant for the mRNA expression of Sox2. The gene expression of established and patient-derived human OS cells cultured under AI conditions to at least the first passage was compared with that of cells cultured in adherent conditions using gene expression profiling with principal component analysis (PCA). There were clear differences in the molecular signatures between adherent and AI cells among established and patient-derived OS cells, although this was less obvious in the established cell lines (Figure 3B). A likely explanation is that the established OS cell lines used are clonally evolved and chemically transformed versions of primary tumor cells, whereas the patient-derived cell lines were grown in culture briefly (<5 passages) prior to analysis. Shortest pathway analysis of the top 300 differentially expressed genes confirmed significant alteration of Runx2, class I HDACs, and Akt (Figure 3C). GeneGo de-novo network generation identified convergence on Wnt/β-catenin, c-myc, and alpha family G-proteins in the molecular signature of AI growth (Additional file 1: Figure S1a and b). These findings pertaining to Runx2 and Wnt/β-catenin signaling are supported through western blot analysis (Additional file 1: Figure S1c). We identified 19 genes that passed false discovery with a corrected p-value less than 0.05 (Table 2). Upregulated genes included those involved in the DNA repair, oncogenic signaling, osteosarcoma pathogenesis, and differentiation, including those specific to mesenchymal lineages [23-26]. Downregulated genes were involved in processes such as proliferation and tumor suppressive functions [27,28]. These results suggest that anoikis-resistant OS cells capable of AI growth have expression changes in key genes that are necessary for cell growth and survival under these conditions.

**Table 2:** **Significantly altered genes in AI OS cells compared to adherent OS cells**

**Upregulated genes**		**Downregulated genes**	
**Gene name**	**Fold increase**	**Gene name**	**Fold decrease**
SOX5	11.30	CCBE1	7.81
PTPRE	5.59	MGLL	7.41
PARP14	3.15	CTGF	6.73
ZNF697	3.09	ABI3BP	5.76
SHC2	3.04	ANXA1	5.50
RUNX2	3.00	DDAH1	5.36
CHST2	2.45	CRIM1	3.66
MTHFSD	2.42	TRNP1	3.64
SH3BP2	2.34	KRT7	3.13
		FHL2	2.92

### Anoikis-resistant OS cells initiate tumors equal to adherent OS cells in both orthotopic and non-orthotopic *in-vivo* xenograft models, but maintain a lower growth rate

When AI and adherent grown human OS cells were injected into athymic nude mice, both initiated tumors at similarly low cell concentrations (Table 3). This was despite our previous finding that AI OS cells had a lower growth rate than adherent cells. This observation was confirmed in both subcutaneous (non-orthotopic) and intratibial (orthotopic) xenograft models. However, cell concentrations as low as 10,000 cells formed tumors intratibially, as opposed to a threshold of 100,000 subcutaneously. These results indicate that anoikis-resistant OS cell subpopulations have a tumorigenic capability similar to that of their adherent counterparts, and that the intratibial microenvironment may be a favorable setting for osteosarcoma tumor formation.

**Table 3:** **Proportion of mice that formed tumors after xenotransplantation of AI or adherent OS cells**

			**Number of cells injected**		
			**1 × 10** ^**4**^	**1 × 10** ^**5**^	**5 × 10** ^**5**^
Subcutaneous					
	mHOS				
		Adherent	0/5	3/5	4/5
		AI	0/5	3/5	3/5
	143B				
		Adherent	0/5	1/5	4/5
		AI	1/5	3/5	5/5
Intratibial					
	mHOS				
		Adherent	6/7	7/7	7/7
		AI	5/7	6/7	7/7
	143B				
		Adherent	5/7	7/7	6/7
		AI	7/7	7/7	6/7

**Figure 4:**
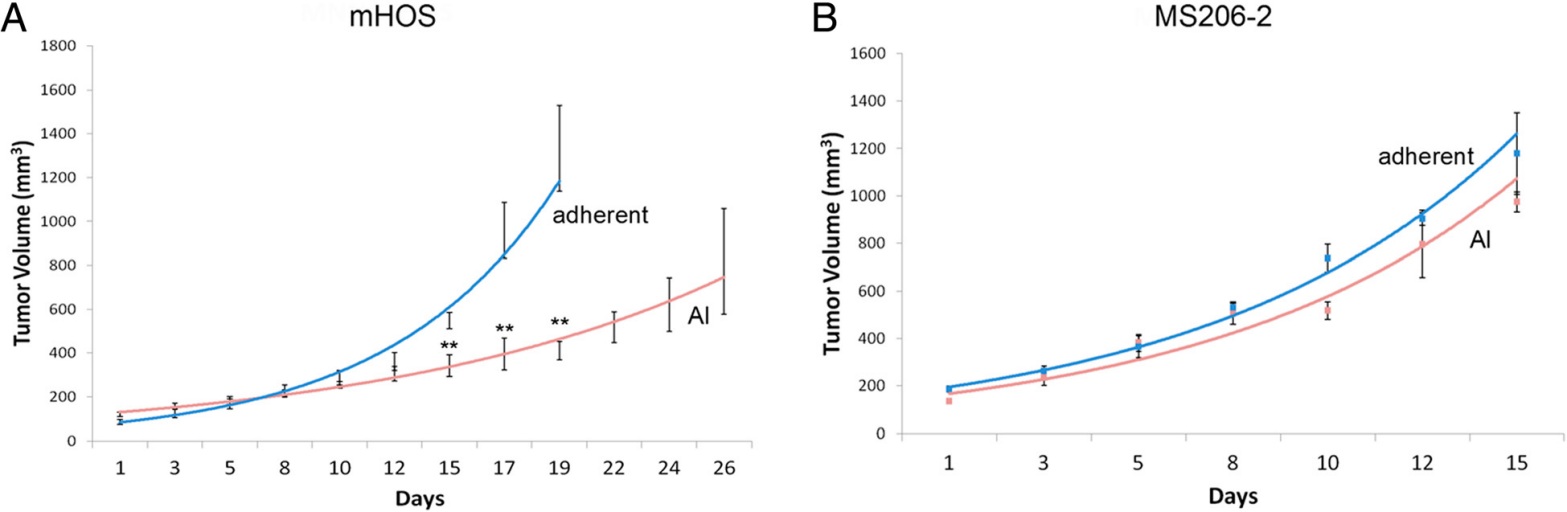
**Altered growth rates of anoikis-resistant AI-grown cells are maintained** ***in-vivo*** **.** mHOS **(A)** and MS206-2 **(B)** cells were under AI (red) and adherent conditions (blue) and were injected intratibially into athymic nude mice. Data shown are trend lines fit to the growth data, with standard deviations for the average tumor volume for that time point. Asterisks indicate statistical significance (**p < 0.01).

To validate whether this alteration in growth rate would hold true in an *in-vivo* setting, 2×10^5^ AI and adherent mHOS cells were injected intratibially into athymic nude mice. Although no differences were noted in tumor initiation rates, there were significant differences in the growth rates once tumors were detected. The tumors derived from AI-grown cells grew more slowly than those from adherent cells (Figure 4). AI-grown patient-derived MS206-2 cells also grew more slowly, although the results were not statistically significant. These results indicate that the slow-growth phenotype of cultured OS cells grown under AI conditions is maintained *in-vivo*.

### Targeted epigenetic therapies decrease OS cell anchorage-independent growth and sensitize to doxorubicin

**Figure 5:**
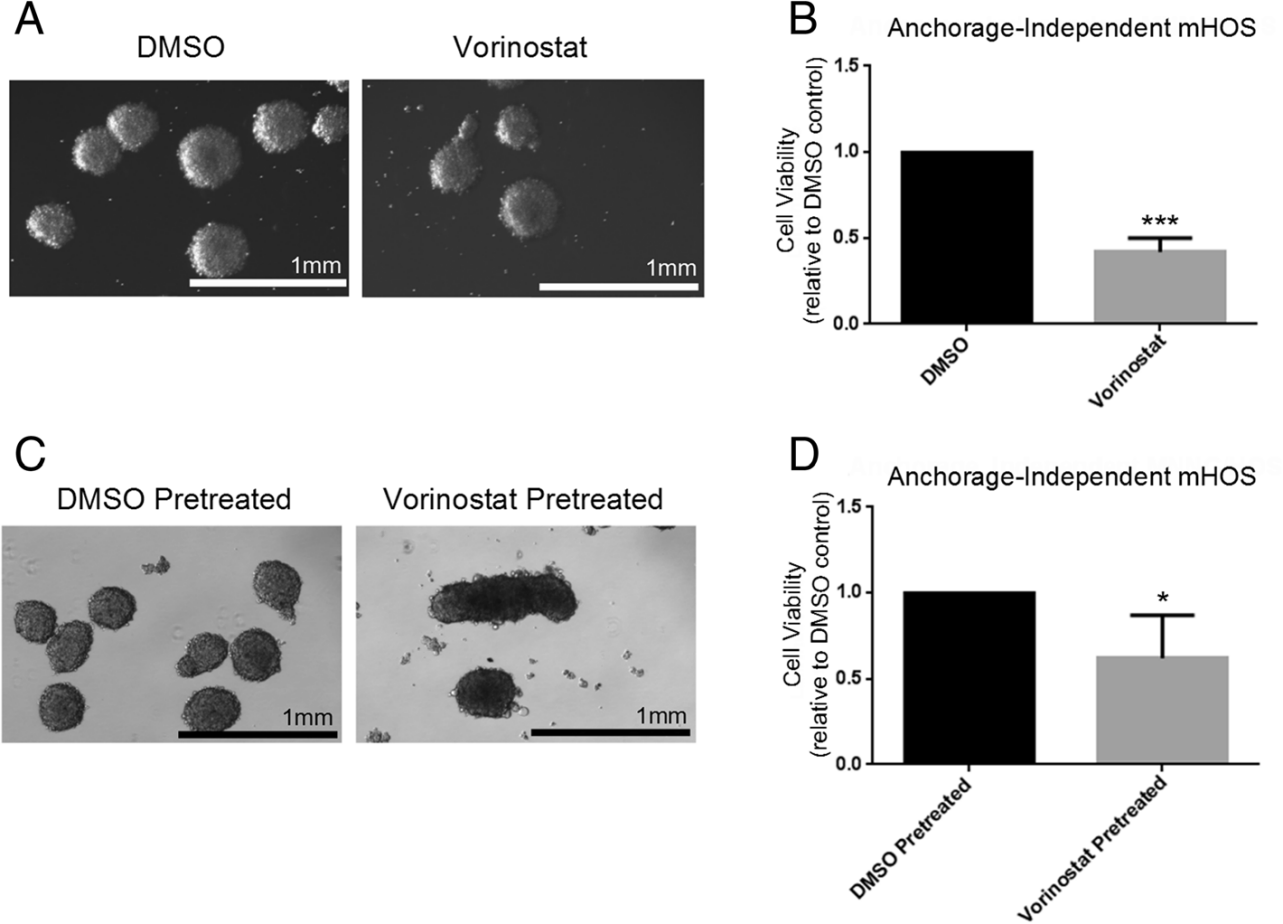
**Vorinostat treatment and preconditioning inhibits anchorage-independent OS cell growth. A**/**B**: mHOS cells were treated with either 2 μM vorinostat or DMSO alone and allowed to grow for 4 days in AI conditions. Vorinostat treatment decreased the number of spheres **(A)**, and resulted in a statistically significant decrease in cell viability **(B)**. **C**/**D**: Adherent mHOS cells were pretreated for 12 days with either 2 μM vorinostat or DMSO alone and subsequently plated and allowed to grow for 4 days in AI conditions in the absence of drug. Vorinostat pretreatment decreased the number of spheres **(C)** and resulted in a significant decrease in cell viability **(D)**. Asterisks indicate statistical significance (*p < 0.05, ***p < 0.001).

The maintenance of phenotypic characteristics of AI-grown OS cells raises the question of possible epigenetic modifications that allow for cell growth and survival under such conditions. Indeed, our observation of c-myc, Akt, β-catenin, and class I HDACs as key components in AI growth strengthens this. Histone deacetylases (HDACs) in many cases are a necessary component of c-myc signaling, and targeted inhibition of HDACs can decrease growth in c-myc overexpressing tumors [29-31]. Treatment of OS cells with 5-azaC induced expression of PTEN, an inhibitor of the PI3K/Akt pathway, while studies in pancreatic cancer demonstrate that 5-azaC treatment reduces proliferation of pancreatic cancer cells through inhibition of Wnt/β-catenin signaling and its downstream target c-myc [32,33]. This literature provides support for HDAC inhibitors and 5-azaC in being able to target a very similar spectrum of signaling pathways that we identified as being altered in anoikis-resistant OS cells. Therefore, we sought to determine the effects on OS cell growth, survival, and the response to doxorubicin under AI conditions of the pan-HDAC inhibitor vorinostat and the DNA methyltransferase inhibitor 5-azaC. AI-grown cells in the presence of 2 μM vorinostat showed a statistically significant decrease in growth after 4 days as relative to DMSO-only controls (Figure 5A, B). We next tested the pretreatment of adherent OS cells with vorinostat and examined their ability to subsequently undergo AI growth. We pretreated adherent OS cells for 12 days with either 2 μM vorinostat or DMSO alone before placing them in ultra-low attachment plates in the absence of drug. Pretreatment with vorinostat decreased the ability of those cells to grow in AI conditions (Figure 5C, D). Neither direct treatment nor pretreatment with vorinostat sensitized AI OS cells to doxorubicin (data not shown). Treatment of adherent mHOS cells with vorinostat similarly decreased cell growth (Additional file 3: Figure S2a). However, vorinostat pretreatment trended towards stimulating adherent cell growth, although this was not statistically significant (Additional file 3: Figure S2a). These results suggest that both direct treatment and prolonged pretreatment with pan-HDAC inhibitors such as vorinostat can effectively mitigate OS cell anoikis resistance.

**Figure 6:**
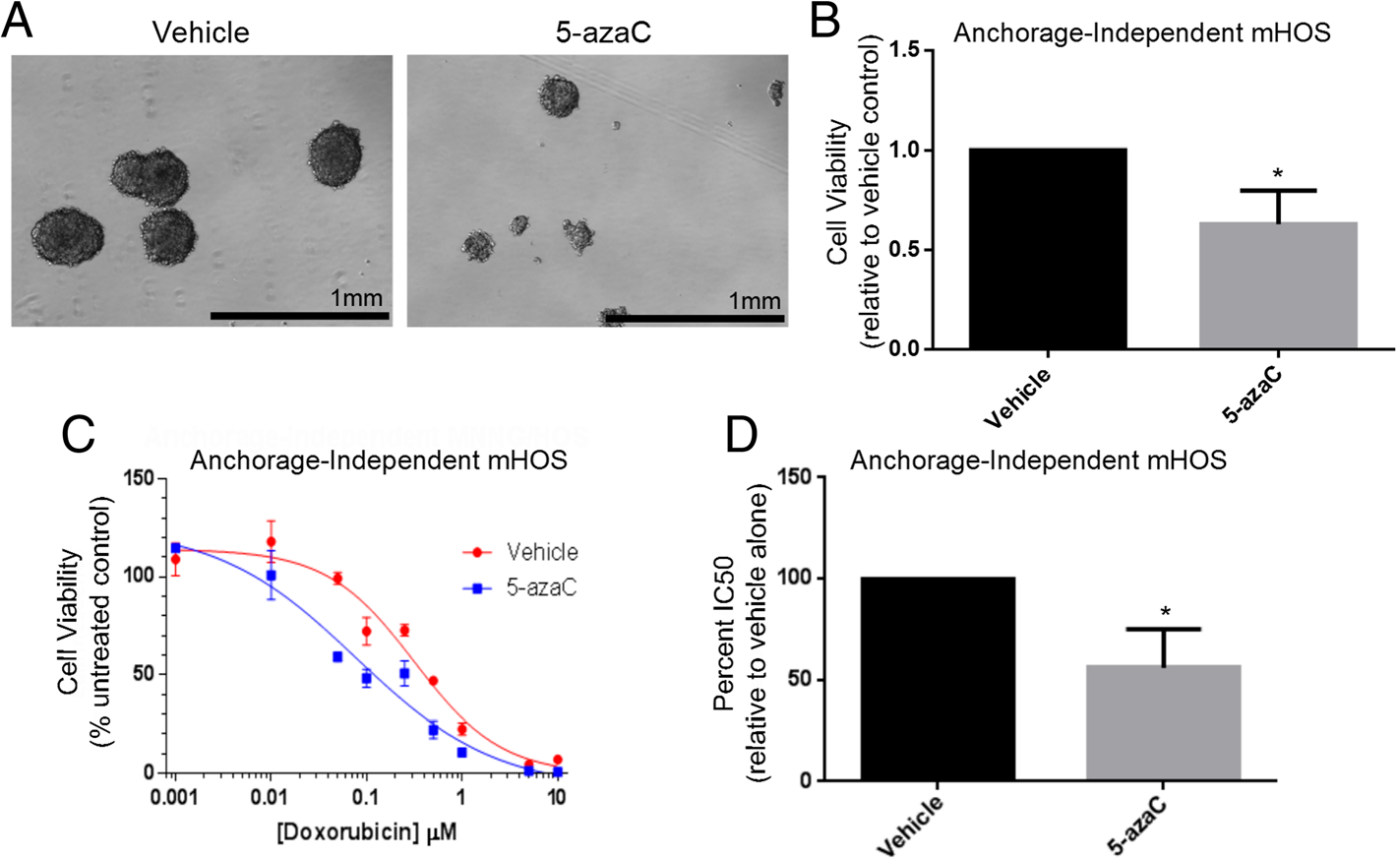
**5-azaC treatment inhibits anchorage-independent OS cell growth while sensitizing to doxorubicin. A/B**: Adherent mHOS cells were treated with either 2 μM 5-azaC or vehicle alone for 24 hours before being plated in AI conditions in the absence of drug and allowed to grow for 4 days. 5-azaC treatment decreased the cells ability to form spheres **(A)** and resulted in a significant decrease in cell viability **(B)**. **C/D**: Adherent mHOS cells were treated with either 2 μM 5-azaC or vehicle alone for 24 hours before being plated in AI conditions in the absence of drug. 24 hours later the cells were treated with one concentration from a serial dilution of doxorubicin, with cell viability being measured after an additional 72 hours. 5-azaC treatment resulted in a left-shift in the doxorubicin dose-response curve **(C)** and a significant decrease in the percent IC_50_ relative to vehicle alone **(D)**. Asterisks indicate statistical significance (*p < 0.05).

We next tested the effect of 5-azaC treatment on OS cell growth and the response to doxorubicin under AI conditions. We treated adherent mHOS cells with 2 μM 5-azaC or vehicle alone for 24 hours before plating in ultra-low attachment plates in the absence of drug. Treatment with 5-azaC decreased the ability of the mHOS cells to grow and survive in AI conditions (Figure 6A, B). Treatment with 5-azaC also resulted in a left-shift in the 72 hr dose-response curve of AI mHOS cells to doxorubicin, indicating that these cells were more sensitive to doxorubicin-mediated toxicity (Figure 6C). This was reflected in a statistically significant decrease in the 72 hr doxorubicin IC_50_ of 5-azaC treated AI cells compared to vehicle treated cells (Figure 6D). Treatment of adherent mHOS cells with 5-azaC trended towards inhibiting cell growth, although this was not statistically significant after the same number of controlled replicates (Additional file 3: Figure S2b). However, no clear difference in the response to doxorubicin was observed in adherent cells treated with 5-azaC (Additional file 3: Figure S2b). These results suggest that 5-azaC treatment can effectively inhibit the growth of anoikis-resistant OS cells while simultaneously sensitizing them to chemotherapy.

## Discussion

The goal of our work was to use gene expression analysis to predict and test targeted therapies to mitigate aspects of OS anoikis resistance. Many of the alterations we noticed on expression profiling can be regulated through epigenetic modifiers, raising the potential for the use of existing FDA-approved epigenetic therapies to target anoikis-resistant OS cells. Proliferation of c-myc overexpressing lymphomas is reduced by HDAC inhibition, and it has been demonstrated in osteosarcoma cells that chromatin-modifying drugs such as vorinostat can increase miRNA expression that leads to decreased c-myc protein and induction of apoptosis [31,34]. 5-azaC treatment inhibits proliferation of pancreatic cancer cells through suppression of β-catenin/c-myc, while studies in OS cells show that 5-azaC treatment increases expression of the PI3K/Akt inhibitor PTEN [32,33]. Our gene expression profiling identified c-myc, β-catenin, Akt and class I HDACs as important contributors to AI growth, thus strengthening the rationale for the use of HDAC inhibitors and 5-azaC in targeting these cell subpopulations. We found that treatment with the HDAC inhibitor vorinostat resulted in decreased AI cell growth. Additionally, pretreatment of adherent cells with vorinostat inhibited their ability to subsequently grow in AI conditions. More work is needed to determine which HDACs are involved, as well as the specific histone acetylation changes taking place that are important for anchorage-independent growth and anoikis resistance. We also found that treatment with 5-azaC reduced AI cell growth and sensitized anoikis-resistant cells to doxorubicin-mediated toxicity. The ability of 5-azaC to sensitize cancer cells to chemotherapy has been observed, particularly in ovarian cancer [35]. Additionally, 5-azaC has been shown to inhibit the Wnt/β-catenin signaling pathway, an important contributor to chemoresistance in human OS [33,36,37]. Future studies include examining changes in levels of DNA methyltransferases as well as determining important genomic loci of DNA methylation in anoikis-resistant OS cells. *In-vivo* assessment of the efficacy of vorinostat and 5-azaC in OS xenografts will also be an area of future study.

Intuitively, acquired chemotherapy resistance to anti-proliferative agents can be greatly affected by the rate of cell division. We noticed that OS cells exhibit a decreased growth rate under AI conditions. Indeed, the literature supports mechanisms of altered growth rate in response to AI growth that involve changes in key cell cycle regulators such as cyclin D1 [38]. Another study in ovarian cancer found that not all cells respond to AI growth with cell cycle arrest, suggesting heterogenous and complex upstream molecular mechanisms [39]. Although more work is required to define the specific mechanism for this growth alteration, we found a variety of candidate mechanisms that would also account for rapid acquisition of chemoresistance beyond a decreased growth rate. We observed increased expression of Sox2 in response to AI growth, which is essential for self-renewal and tumorigenicity in OS tumor-initiating cells [40]. Sox2 has also been implicated in conveying chemoresistance to cancer stem cell-like cells in other cancers through ABCG2 expression levels [41]. Comparison of the gene expression profiles of AI and adherent OS cells from freshly harvested patient tumors confirms a common adaptation process underlying resistance to anoikis and chemotherapy. Unbiased molecular pathway analysis of the top 300 differentially expressed genes identified consistent signaling convergence on Wnt signaling, Runx2, c-myc, and components of histone modification. Aberrant Wnt signaling contributes to OS tumorigenesis, and inhibition of Wnt signaling effectively sensitizes OS cells to chemotherapy [36,37,42]. c-myc overexpression is transformative to bone marrow stromal cells and conveys invasive properties to OS cells and resistance to cisplatin in other cancer cell lines [43-45], while many signaling events that are mediated by c-myc require functioning HDACs [29,30]. Furthermore, increased expression of Runx2 can suppress proliferation and chemosensitivity of OS cells and has been shown to inhibit p53-dependent apoptosis together with HDACs in response to DNA damage [46-48]. Taken together, these pathways represent candidate contributors to the chemoresistance of anchorage-independent human OS cells observed in our study beyond altered cell growth. It is worth noting that the expression profile of the chemically transformed, highly clonal established OS cells showed less dramatic changes in the anoikis-resistant gene signature. There was marked agreement between the findings of the pathway analysis and the individual differentially expressed genes identified, particularly as they relate to overall processes such as cell growth and oncogenic signaling. In fact, Runx2 was identified in both analyses as a significant contributor to OS anchorage-independent growth. Future work will include interrogation of the pathway and gene alterations identified to determine their specific contributions towards the phenotypic consequences of OS anoikis resistance.

We found that adherent and AI-grown OS cells were equally tumorigenic in subcutaneous and intratibial settings; the latter is arguably more similar to the osteosarcoma tumor microenvironment. Prior work shows that AI-grown OS cells are more capable of tumor initiation, and the reasons for this difference are unclear [12,14]. Though we were unable to demonstrate a greater tumor-initiating potential, cells cultivated in AI conditions maintain their decreased growth rate relative to adherent OS cells *in-vivo*. It will be important in future studies to examine metastatic rates between adherent and AI grown OS cells. These data indicate that the phenotypic behavior of AI-grown cells is not a transient finding.

The cultivation of human osteosarcoma cells through anchorage-independent growth alters the growth rate, promotes chemotherapy resistance, and produces a distinct molecular signature both *in-vitro* and *in-vivo*. We show that acquisition of anoikis resistance in human osteosarcoma subpopulations through anchorage-independent growth results in a rapid development of chemoresistance and altered growth rate, mirroring the early stages of latent metastasis. Targeting epigenetic regulation of this process may be a viable therapeutic strategy.

## Additional files

Additional file 1: Figure S1. Genego and shortest pathway analysis of AI and adherent OS cells. A: Genego molecular signature identifies Wnt/β-catenin and the G-protein alpha family as significant nodes in AI OS cell growth. B: GeneGO molecular pathway analysis shows significant convergence on c-Myc in the AI expression signature. C: Support for altered Runx2 and Wnt/β-catenin signaling through western blot analysis of adherent and anoikis-resistant (p2 sphere) mHOS and MG-63 human OS cells. Additional file 2: Table S1. Top 300 gene list by GeneGo analysis. Additional file 3: Figure S2. Vorinostat and 5-azaC treatment of adherent OS cells. A: mHOS cells were treated with either 2 μM vorinostat or DMSO alone and allowed to grow for 4 days in adherent conditions, which resulted in a statistically significant decrease in cell viability (left panel). Adherent mHOS cells were pretreated for 12 days with either 2 μM vorinostat or DMSO alone and subsequently plated and allowed to grow for 4 days in adherent conditions in the absence of drug. Vorinostat pretreatment trended towards stimulating adherent OS cell growth, although this was not statistically significant (right panel). B: Adherent mHOS cells were treated with either 2 μM 5-azaC or vehicle alone for 24 hours before being plated in adherent conditions in the absence of drug and allowed to grow for 4 days. 5-azaC trended towards inhibiting cell growth, although this was not statistically significant after the same number of controlled replicates (left panel). Adherent mHOS cells were treated with either 2 μM 5-azaC or vehicle alone for 24 hours before being plated in adherent conditions in the absence of drug. 24 hours later the cells were treated with one concentration from a serial dilution of doxorubicin, with cell viability being measured after an additional 72 hours. 5-azaC treatment did not alter the doxorubicin dose-response curve (middle panel), and there was no significant difference in the percent IC_50_ relative to vehicle alone (right panel). Asterisks indicate statistical significance (***p < 0.001, NS; not significant (p > 0.05)).
