# Supplementary figures and images for: Anoikis-resistant subpopulations of human osteosarcoma display significant chemoresistance and are sensitive to targeted epigenetic therapies predicted by expression profiling
Source: J Transl Med. 2015 Apr 2;13:110. doi: 10.1186/s12967-015-0466-4 (PMC4419490; doi:10.1186/s12967-015-0466-4)

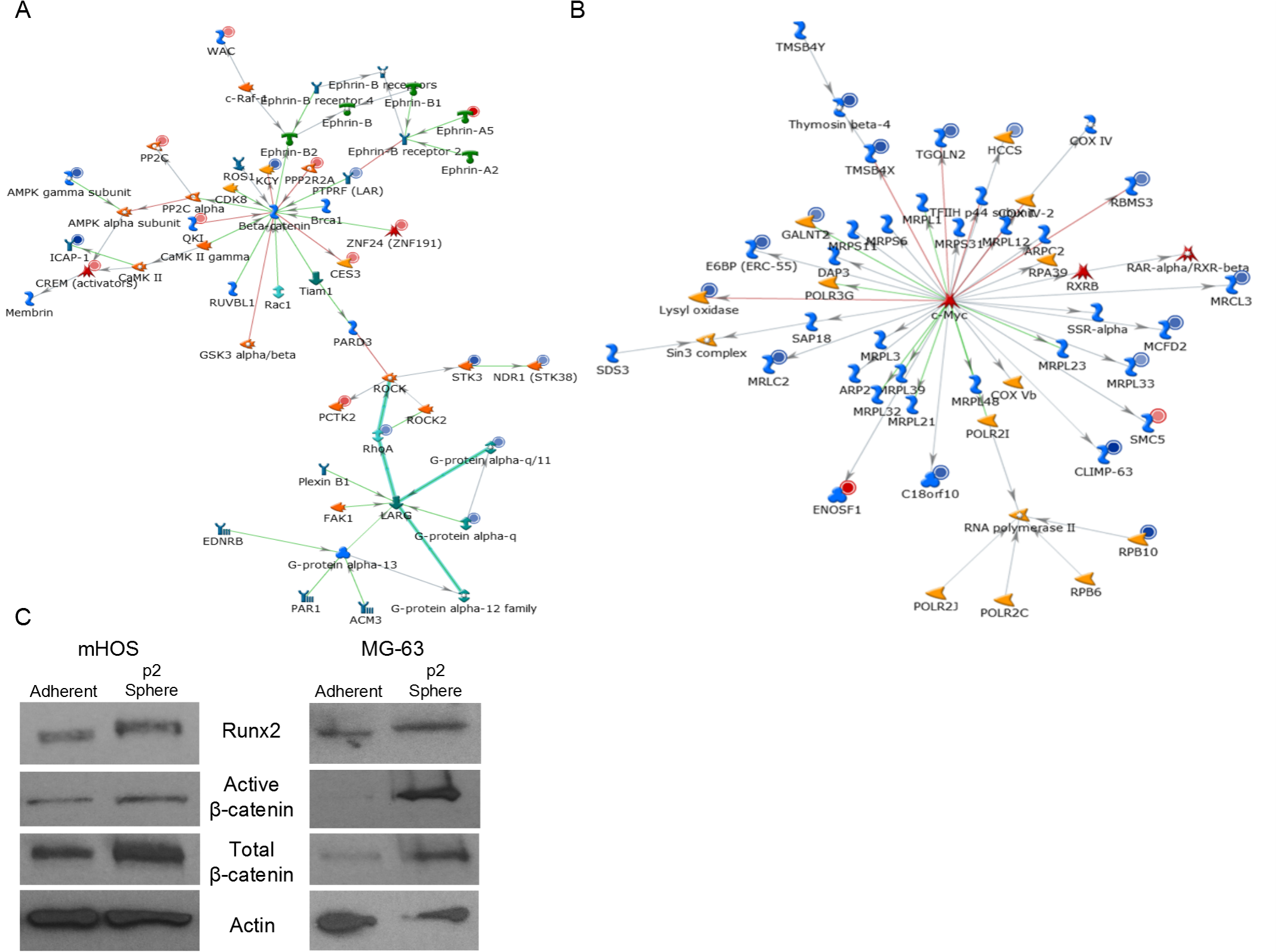

Supplement: Additional file 1: Figure S1. — Genego and shortest pathway analysis of AI and adherent OS cells. A: Genego molecular signature identifies Wnt/β-catenin and the G-protein alpha family as significant nodes in AI OS cell growth. B: GeneGO molecular pathway analysis shows significant convergence on c-Myc in the AI expression signature. C: Support for altered Runx2 and Wnt/β-catenin signaling through western blot analysis of adherent and anoikis-resistant (p2 sphere) mHOS and MG-63 human OS cells. [file 12967_2015_466_MOESM1_ESM.tiff]

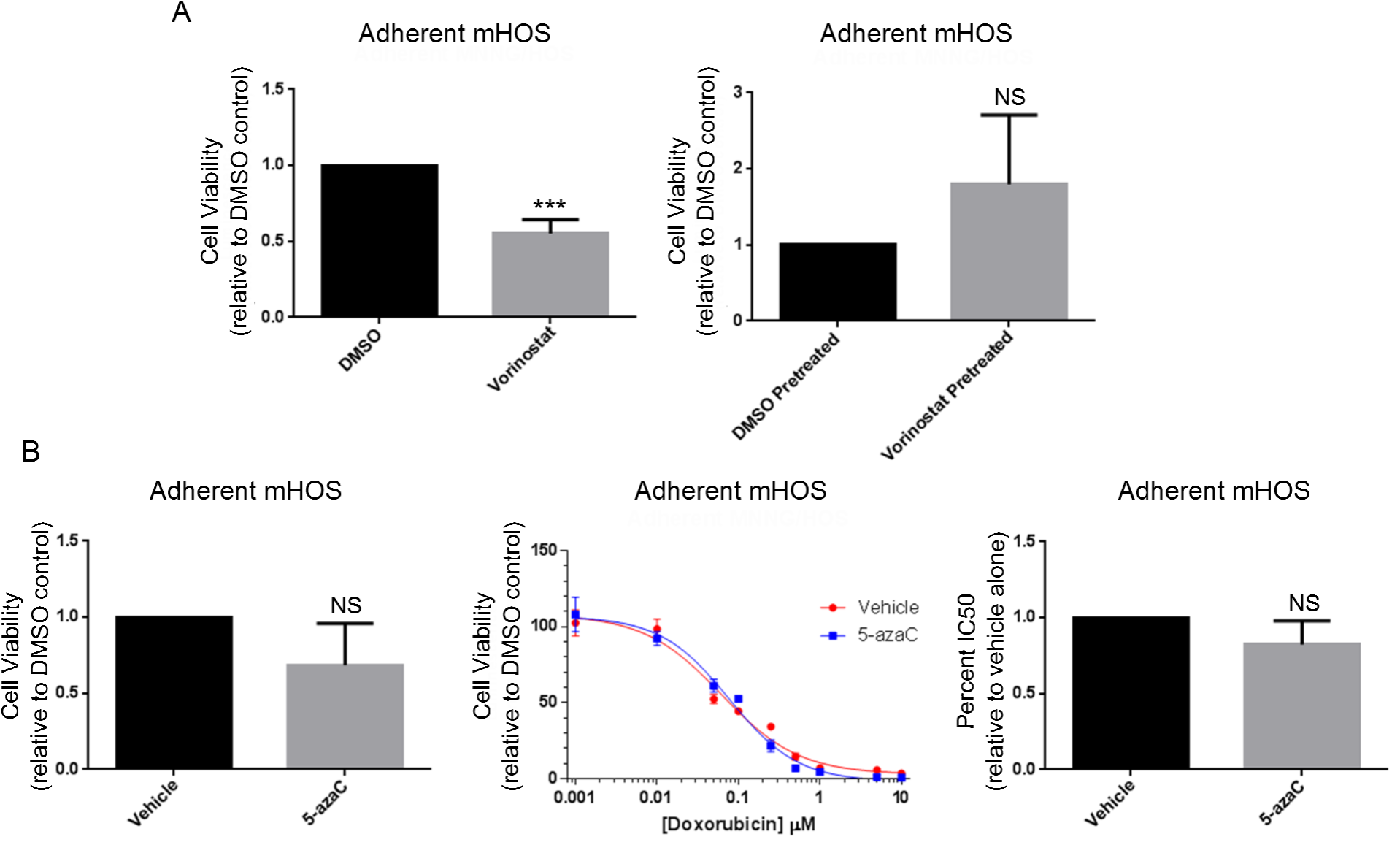

Supplement: Additional file 3: Figure S2. — Vorinostat and 5-azaC treatment of adherent OS cells. A: mHOS cells were treated with either 2 μM vorinostat or DMSO alone and allowed to grow for 4 days in adherent conditions, which resulted in a statistically significant decrease in cell viability (left panel). Adherent mHOS cells were pretreated for 12 days with either 2 μM vorinostat or DMSO alone and subsequently plated and allowed to grow for 4 days in adherent conditions in the absence of drug. Vorinostat pretreatment trended towards stimulating adherent OS cell growth, although this was not statistically significant (right panel). B: Adherent mHOS cells were treated with either 2 μM 5-azaC or vehicle alone for 24 hours before being plated in adherent conditions in the absence of drug and allowed to grow for 4 days. 5-azaC trended towards inhibiting cell growth, although this was not statistically significant after the same number of controlled replicates (left panel). Adherent mHOS cells were treated with either 2 μM 5-azaC or vehicle alone for 24 hours before being plated in adherent conditions in the absence of drug. 24 hours later the cells were treated with one concentration from a serial dilution of doxorubicin, with cell viability being measured after an additional 72 hours. 5-azaC treatment did not alter the doxorubicin dose-response curve (middle panel), and there was no significant difference in the percent IC50 relative to vehicle alone (right panel). Asterisks indicate statistical significance (***p < 0.001, NS; not significant (p > 0.05)). [file 12967_2015_466_MOESM3_ESM.tiff]
